# *Boesenbergia stenophylla*-Derived Stenophyllol B Exerts Antiproliferative and Oxidative Stress Responses in Triple-Negative Breast Cancer Cells with Few Side Effects in Normal Cells

**DOI:** 10.3390/ijms24097751

**Published:** 2023-04-24

**Authors:** Min-Yu Lee, Jun-Ping Shiau, Jen-Yang Tang, Ming-Feng Hou, Phoebe Sussana Primus, Chai-Lin Kao, Yeun-Mun Choo, Hsueh-Wei Chang

**Affiliations:** 1 Graduate Institute of Medicine, College of Medicine, Kaohsiung Medical University, Kaohsiung 80708, Taiwan; u111500005@gap.kmu.edu.tw (M.-Y.L.); mifeho@kmu.edu.tw (M.-F.H.); 2 Division of Breast Oncology and Surgery, Department of Surgery, Kaohsiung Medical University Hospital, Kaohsiung Medical University, Kaohsiung 80708, Taiwan; 1060526@kmuh.org.tw; 3 School of Post-Baccalaureate Medicine, Kaohsiung Medical University, Kaohsiung 80708, Taiwan; reyata@kmu.edu.tw; 4 Department of Radiation Oncology, Kaohsiung Medical University Hospital, Kaohsiung Medical University, Kaohsiung 80708, Taiwan; 5 Department of Biomedical Science and Environmental Biology, College of Life Science, Kaohsiung Medical University, Kaohsiung 80708, Taiwan; 6 Department of Chemistry, Faculty of Science, University of Malaya, Kuala Lumpur 50603, Malaysia; phoebe18@siswa.um.edu.my; 7 Department of Medicinal and Applied Chemistry, Drug Development and Value Creation Research Center, Kaohsiung Medical University, Kaohsiung 80708, Taiwan; clkao@kmu.edu.tw; 8 Center for Cancer Research, Kaohsiung Medical University, Kaohsiung 80708, Taiwan

**Keywords:** *Boesenbergia* plants, triple-negative breast cancer, oxidative stress, apoptosis, DNA damage

## Abstract

Triple-negative breast cancer (TNBC) is insensitive to target therapy for non-TNBC and needs novel drug discovery. Extracts of the traditional herb *Boesenbergia* plant in Southern Asia exhibit anticancer effects and contain novel bioactive compounds but merely show cytotoxicity. We recently isolated a new compound from *B. stenophylla*, stenophyllol B (StenB), but the impact and mechanism of its proliferation-modulating function on TNBC cells remain uninvestigated. This study aimed to assess the antiproliferative responses of StenB in TNBC cells and examine the drug safety in normal cells. StenB effectively suppressed the proliferation of TNBC cells rather than normal cells in terms of an ATP assay. This preferential antiproliferative function was alleviated by pretreating inhibitors for oxidative stress (*N*-acetylcysteine (NAC)) and apoptosis (Z-VAD-FMK). Accordingly, the oxidative-stress-related mechanisms were further assessed. StenB caused subG1 and G2/M accumulation but reduced the G1 phase in TNBC cells, while normal cells remained unchanged between the control and StenB treatments. The apoptosis behavior of TNBC cells was suppressed by StenB, whereas that of normal cells was not suppressed according to an annexin V assay. StenB-modulated apoptosis signaling, such as for caspases 3, 8, and 9, was more significantly activated in TNBC than in normal cells. StenB also caused oxidative stress in TNBC cells but not in normal cells according to a flow cytometry assay monitoring reactive oxygen species, mitochondrial superoxide, and their membrane potential. StenB induced greater DNA damage responses (γH2AX and 8-hydroxy-2-deoxyguanosine) in TNBC than in normal cells. All these StenB responses were alleviated by NAC pretreatment. Collectively, StenB modulated oxidative stress responses, leading to the antiproliferation of TNBC cells with little cytotoxicity in normal cells.

## 1. Introduction

Breast cancer is the primary reason for cancer death in women, showing a 30% incidence of female cancer [1]. The incidence of breast cancer annually increases by 0.5%. Generally, most breast cancer cells present differential amounts of three significant biomarkers, i.e., the estrogen receptor (ER), progesterone receptor (PR), and human epidermal growth factor receptor 2 (HER2) [2]. Some breast cancer cells (10–15%) lacking these three markers are named triple-negative breast cancer (TNBC) cells [3] and are unsuitable for treatment with the typical targeted therapy for breast cancer. TNBC generally occurs in patients at a younger age, and they have a higher recurrence than non-TNBC patients, leading to TNBC patients having a higher death rate [4,5]. New TNBC drug developments are needed more immediately than non-TNBC ones. Moreover, some clinical drugs for breast cancer chemotherapy are occasionally accompanied by severe side effects [6]. Identifying prospective antibreast drugs with low side effects is still important.

The *Boesenbergia* plants (Zingiberaceae family), which are perennial rhizomatous herbs, have approximately 70 species and are widely distributed in Thailand, Malaysia, Brunei, Indonesia, and the Philippines. About one-third of *Boesenbergia* plants are indigenous to Borneo [7,8]. Moreover, several biological effects of the extracts derived from *Boesenbergia* plants have been reported, including antiulcerogenic (*B. rotunda*) [9,10], wound-healing (*B. rotunda*) [11], antibacterial (*B. rotunda*) [12,13], anti-SARS-CoV-2 (*B. rotunda*) [14], and antioxidant (*B. rotunda* [15] and *B. pulcherrima* [16]) properties.

The anticancer effects of *Boesenbergia* plant extracts, such as *B. rotunda*, *B. pulchella* var. attenuata, and *B. armeniaca* [17], against breast, ovarian, colon, and cervical cancer cells have been reported, but studies have mainly focused on cytotoxicity. Additionally, the anticancer effects of several bioactive compounds from *B. pandurate* [18,19,20] and *B. rotunda* [21,22] have been reported in breast, colon, and nasopharyngeal cancer cells. This increasing trend in antineoplastic bioactive drug candidates warrants the identification of more Boesenbergia-plant-derived natural products for anticancer treatment.

*B. stenophylla*, one of the *Boesenbergia* plants from Borneo, exhibits medicinal properties, such as protecting from convulsions, suppressing intoxication, reducing cough, and alleviating food poisoning [7,23]. The chemical properties and cellular impacts of *B. stenophylla* remain unclear. Its chemical constituents have been analyzed for use in essential oils without providing cytotoxicity information for cancer cells [7]. The anticancer effects of *B. stenophylla* have rarely been assessed. Recently, three new *B. stenophylla*-derived compounds, such as stenophyllol A, stenophyllol B (StenB), and stenophyllol C, were reported in our previous study [24]. Except for purification and chemical characterization, only the antiproliferation effects of stenophyllol A were reported in neuroblastoma cells.

This study aimed to assess the antiproliferation effects of StenB through the example of TNBC cells. The safety of StenB was examined via normal breast cells. Moreover, the potential mechanism of StenB for exerting anticancer effects on breast cancer cells was investigated.

## 2. Results

### 2.1. Antiproliferation Response to StenB (TNBC vs. Normal Cells)

**Figure 1:**
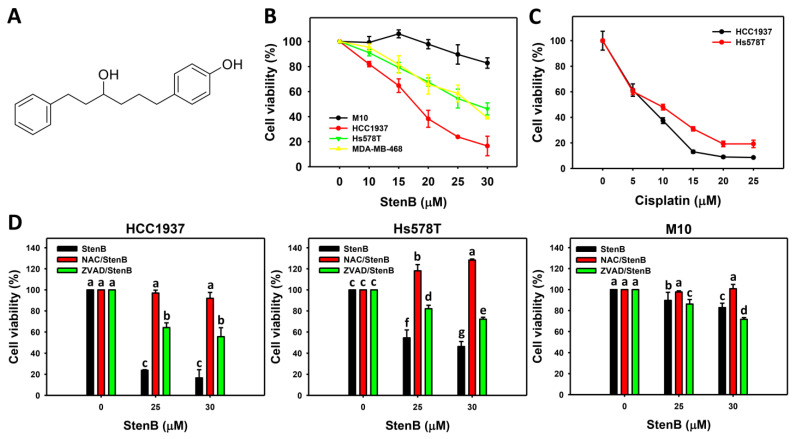
Effects of StenB on the viability of TNBC and M10 cells. (**A**) Structure. (**B**) Cell viability of StenB. TNBC and normal (M10) cells were treated with StenB for 48 h. Cell viability was assessed by ATP assay. (**C**) Cell viability of cisplatin. TNBC cells were treated with cisplatin for 48 h. (**D**) Cell viability of NAC and/or StenB. Cells received NAC (10 mM, 1 h) or ZVAD (100 μM, 2 h) pretreatment and/or StenB (0, 25, and 30 μM) post-treatment for 48 h. Data = mean ± SD (*n* = 3). Each treatment was assigned the connecting letters by software to judge significance. Connecting letters for different treatments without overlapping differed significantly (*p* < 0.05). (**D**) (Hs578T cells) shows that StenB at 0, 25, and 30 μM, which were assigned with “c, f, and g” by JMP statistical software, indicates significant differences as nonoverlapping letters appeared. StenB, NAC/StenB, and ZVAD/StenB of Hs578T cells at 25 or 30 μM, which were assigned with “f, b, and d” or “g, a, and e”, also differ significantly.

Following the increase in the StenB (Figure 1A) concentration, the cell viability of breast cancer cells (HCC1937 and Hs587T) was dramatically decreased (Figure 1B), whereas there was little cell viability impact of StenB on normal breast cells H184B5F5/ M10 (M10). Consequently, StenB effectively demonstrated antiproliferative effects against TNBC cells, but exhibited low cytotoxic effects against normal cells. For comparison, cisplatin showed to be more sensitive to TNBC cells compared to StenB (Figure 1C).

Additionally, the potential role of oxidative stress in StenB-induced antiproliferation was evaluated by the *N*-acetylcysteine (NAC) pretreatment, which is an ROS scavenger for reducing oxidative stress. The NAC pretreatment/StenB post-treatment (NAC/StenB) demonstrated higher cell viability in TNBC cells than the StenB treatment (Figure 1D). Accordingly, StenB-inducing oxidative stress incurs the antiproliferation of TNBC cells. Moreover, the potential role of apoptosis in StenB-induced antiproliferation was evaluated by the Z-VAD-FMK (ZVAD) pretreatment, which is a pan-caspase inhibitor. ZVAD/StenB demonstrated higher cell viability in TNBC cells than the StenB treatment (Figure 1D). Accordingly, StenB incurs the antiproliferation of TNBC cells by modulating oxidative stress and apoptosis.

### 2.2. Cell Cycle Response to StenB (TNBC vs. Normal Cells)

**Figure 2:**
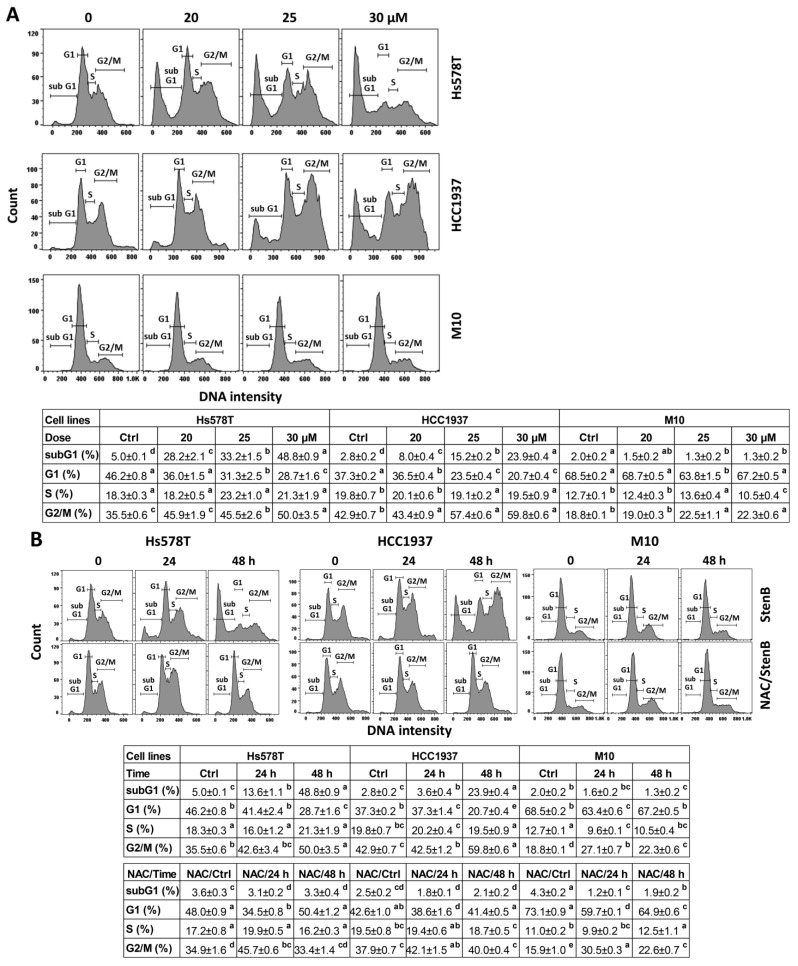
Effects of StenB on cell cycle of TNBC and M10 cells. (**A**) Cell cycle. TNBC and normal (M10) cells were treated with StenB for 48 h. (**B**) Cell cycle of NAC and/or StenB. Cells received NAC pretreatment (10 mM, 1 h) and/or StenB (30 μM) post-treatment for 0, 24, and 48 h. Data = mean ± SD (*n* = 3). Each treatment was assigned the connecting letters by software to judge significance. Different letters in the up-right side of the mean ± SD indicate significant differences (*p* < 0.05).

Abnormal cell cycle progression may contribute to drug-induced antiproliferation. Following StenB treatment, the subG1 (%) of TNBC cells (HCC1937 and Hs578T) (Figure 2A) was raised, showing a higher subG1 (%) of TNBC cells than normal cells. Consequently, StenB effectively incurred an apoptosis-like subG1 increase in TNBC cells but not in normal cells. Moreover, StenB decreased the G1 (%) of TNBC cells and caused the G2/M arrest of TNBC cells, particularly at 25 and 30 μM. In comparison, normal cells maintained a similar cell cycle distribution for the control and StenB treatment (25 to 30 μM).

Additionally, the potential role of oxidative stress in the StenB-induced subG1 increase and G2/M arrest was evaluated by NAC. For example, in the 48 h experiment, NAC/StenB demonstrated a lower subG1 (%), higher G1 (%), and lower G2/M (%) of TNBC cells than the StenB treatment (Figure 2B). Accordingly, StenB incurs a subG1 increase and G2/M arrest of TNBC cells by modulating oxidative stress.

### 2.3. Annexin V Response to StenB (TNBC vs. Normal Cells)

**Figure 3:**
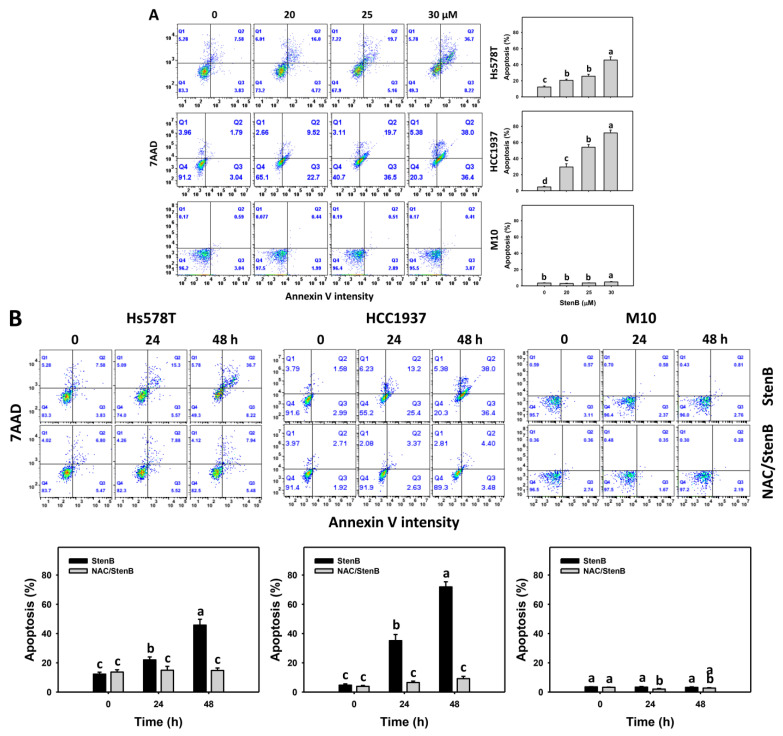
Effects of StenB on annexin V intensity in TNBC and M10 cells. (**A**) Annexin V levels. TNBC and normal (M10) cells were treated with StenB for 48 h. (**B**) Annexin V levels of NAC and/or StenB. Cells received NAC pretreatment (10 mM, 1 h) and/or StenB (30 μM) post-treatment for 0, 24, and 48 h. The apoptosis proportions, showing annexin V (+)/7AAD (+ or −) intensity, were assigned to apoptotic (+) cells. Data = mean ± SD (*n* = 3). Each treatment was assigned the connecting letters by software to judge significance. Different letters above bars indicate significant differences (*p* < 0.05).

The phenomena of the increase in subG1 justifies an additional examination of apoptosis, such as with an annexin V/7-aminoactinmycin D (7AAD) assessment. Following the StenB treatment, the level of annexin V (+)(%) in TNBC cells (HCC1937 and Hs578T) (Figure 3A) was raised, showing a higher annexin V (+)(%) level in TNBC cells than normal cells. Consequently, StenB effectively incurred the apoptosis of TNBC cells but not of normal cells.

Additionally, the potential role of oxidative stress in the StenB-induced annexin V (+)(%) increase was evaluated by NAC. StenB demonstrated higher annexin V (+)(%) levels in TNBC cells than the NAC/StenB treatment (Figure 3B). Accordingly, StenB incurs the apoptosis of TNBC cells by modulating oxidative stress.

### 2.4. Caspase 3 Responses to StenB (TNBC vs. Normal Cells)

**Figure 4:**
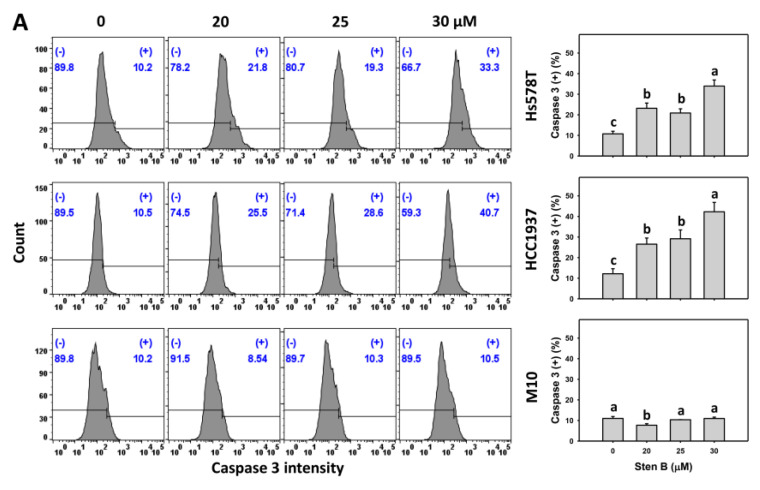

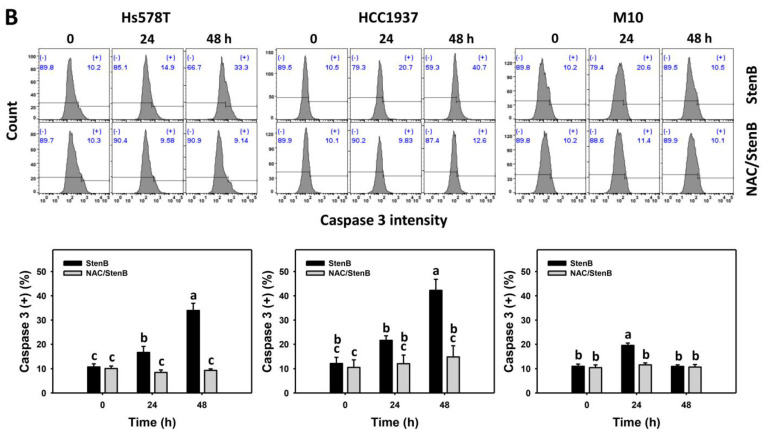
Effects of StenB on caspase 3 intensity in TNBC and M10 cells. (**A**) Caspase 3 activity. TNBC and normal (M10) cells were treated with StenB for 48 h. (**B**) Caspase 3 activity of NAC and/or StenB. Cells received NAC pretreatment (10 mM, 1 h) and/or StenB (30 μM) post-treatment for 0, 24, and 48 h. The proportions marked with (+) were assigned to caspase 3 (+) cells. Data = mean ± SD (*n* = 3). Each treatment was assigned the connecting letters by software to judge significance. Different letters above bars indicate significant differences (*p* < 0.05).

To fully support the results of apoptosis induction (annexin V) by StenB, apoptosis signaling via caspase 3 was examined. Following the StenB treatment, the caspase 3 (+)(%) levels in TNBC cells (HCC1937 and Hs578T) (Figure 4A) were raised, showing higher Cas 3 (+)(%) levels in TNBC cells than in normal cells. Consequently, StenB effectively incurs caspase 3 activation of apoptosis signaling in TNBC cells but not in normal cells.

Additionally, the potential role of oxidative stress in the StenB-induced caspase 3 (+)(%) increase was evaluated by NAC. StenB demonstrated higher caspase 3 (+)(%) levels in TNBC cells than the NAC/StenB treatment (Figure 4B). Accordingly, StenB incurs the activation of caspase 3 in apoptosis signaling in TNBC cells by modulating oxidative stress.

### 2.5. Caspase 8 and 9 Responses to StenB (TNBC vs. Normal Cells)

**Figure 5:**
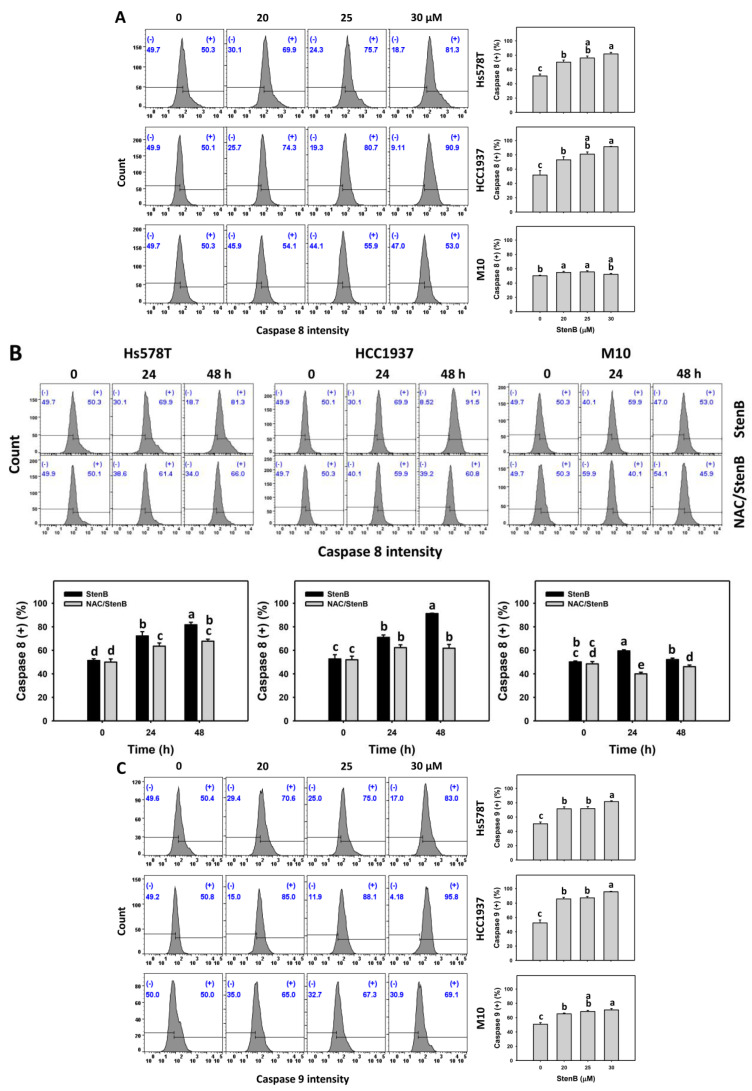

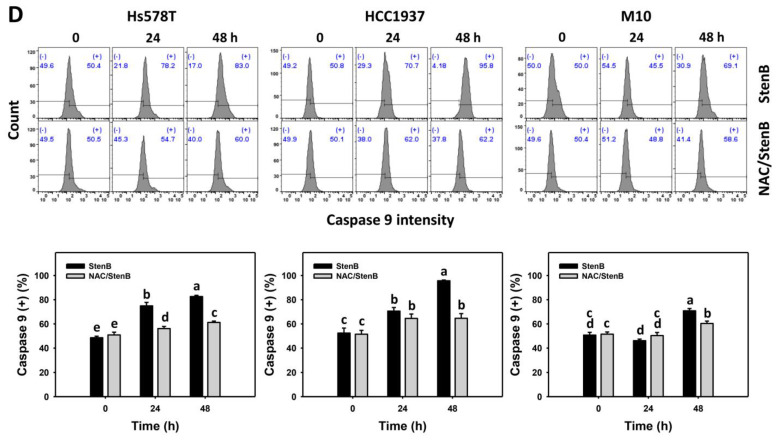
Effects of StenB on caspase 8 and 9 intensity in TNBC and M10 cells. (**A**,**C**) Caspase 8 and Caspase 9 activity. TNBC and normal (M10) cells were treated with StenB for 48 h. (**B**,**D**) Caspase 8 and Caspase 9 activity of NAC and/or StenB. Cells received NAC pretreatment (10 mM, 1 h) and/or StenB (30 μM) post-treatment for 0, 24, and 48 h. The proportions marked with (+) were assigned to caspase 8 and 9 (+) cells. Data = mean ± SD (*n* = 3). Each treatment was assigned the connecting letters by software to judge significance. Different letters above bars indicate significant differences (*p* < 0.05).

To further investigate the upstream response of caspase 3 activation by StenB, extrinsic and intrinsic apoptosis signaling via caspases 8 and 9 was examined. Following the StenB treatment, the levels of caspases 8 and 9 (+)(%) in TNBC cells (HCC1937 and Hs578T) (Figure 5A) were raised, showing higher levels of caspases 8 and 9 (+)(%) in TNBC cells than in normal cells. Consequently, StenB effectively incurs the activation of caspases 8 and 9 in apoptosis signaling in TNBC cells but not in normal cells.

Additionally, the potential role of oxidative stress in the increase in the levels of StenB-induced caspases 8 and 9 (+)(%)was evaluated by NAC. StenB demonstrated higher levels of caspases 8 and 9 (+)(%) in TNBC cells than the NAC/StenB treatment (Figure 5B). Accordingly, StenB incurs the activation of caspases 8 and 9 in apoptosis signaling in TNBC cells by modulating oxidative stress.

### 2.6. Reactive Oxygen Species (ROS) and Mitochondrial Superoxide (MitoSOX) Responses to StenB (TNBC vs. Normal Cells)

**Figure 6:**
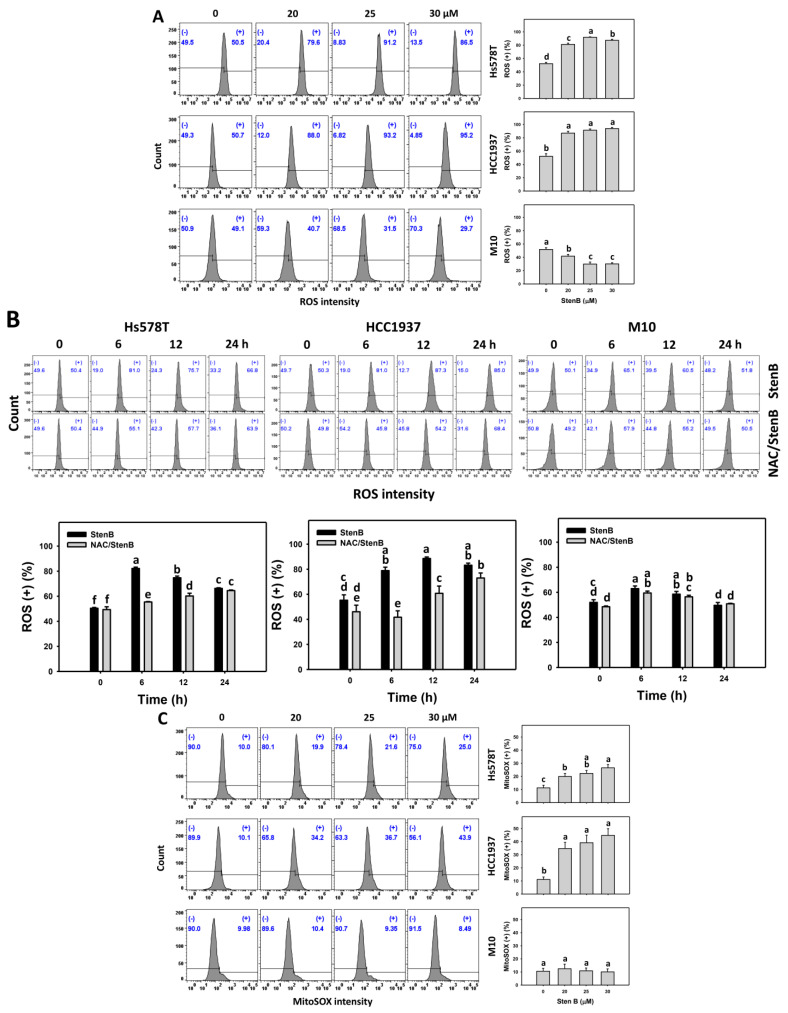

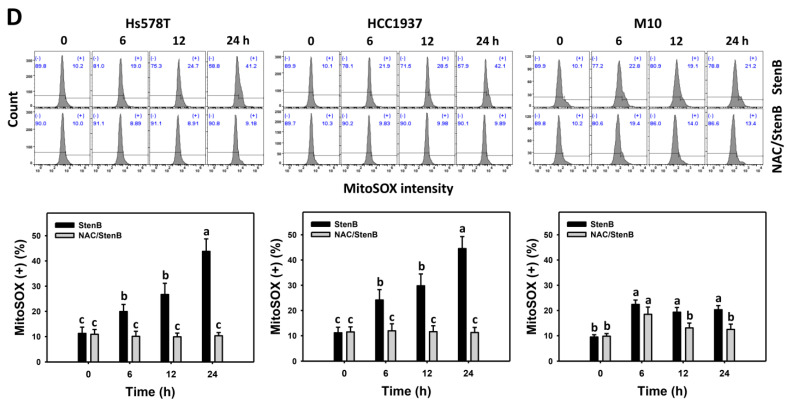
Effects of StenB on ROS and MitoSOX intensity in TNBC and M10 cells. (**A**,**C**) ROS and MitoSOX levels. TNBC and normal (M10) cells were treated with StenB for 48 h. (**B**,**D**) ROS and MitoSOX levels of NAC and/or StenB. Cells received NAC pretreatment (10 mM, 1 h) and/or StenB (30 μM) post-treatment for 0, 6, 12, and 24 h. The proportions marked with (+) were assigned to ROS and MitoSOX (+) cells. Data = mean ± SD (*n* = 3). Each treatment was assigned the connecting letters by software to judge significance. Different letters above bars indicate significant differences (*p* < 0.05).

Following the StenB treatment, the ROS and MitoSOX (+)(%) levels in TNBC cells (HCC1937 and Hs578T) (Figure 6A,C) were raised, showing higher ROS and MitoSOX (+)(%) levels in TNBC cells than in normal cells. Consequently, StenB effectively incurs cellular and mitochondrial oxidative stress in TNBC cells but not in normal cells.

Additionally, the potential role of oxidative stress in the StenB-induced ROS and MitoSOX (+)(%) increase was evaluated by NAC. StenB demonstrated higher ROS and MitoSOX (+)(%) levels in TNBC cells than the NAC/StenB treatment (Figure 6B,D). Accordingly, StenB incurs cellular and mitochondrial oxidative stress in TNBC cells.

### 2.7. Mitochondrial Membrane Potential (MMP) Response to StenB (TNBC vs. Normal Cells)

**Figure 7:**
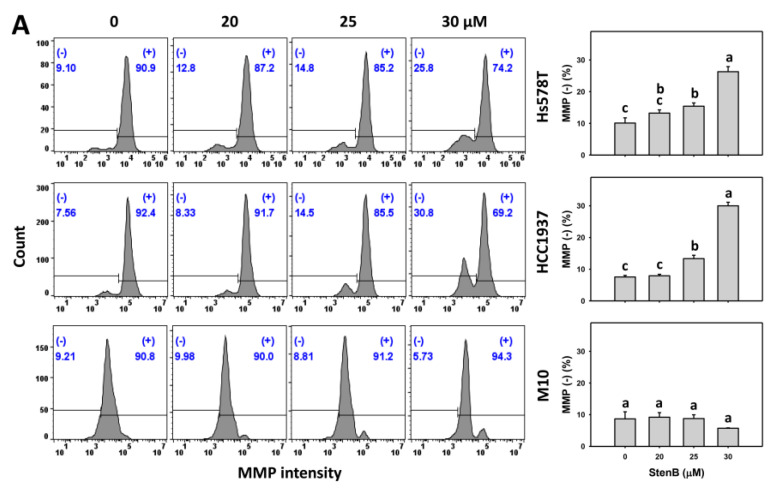

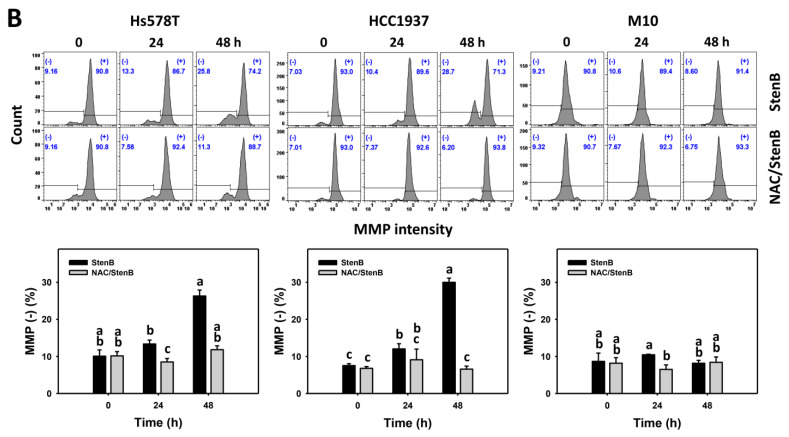
Effects of StenB on MMP (−) intensity in TNBC and M10 cells. (**A**) MMP levels. TNBC and normal (M10) cells were treated with StenB for 48 h. (**B**) MMP levels of NAC and/or StenB. Cells received NAC pretreatment (10 mM, 1 h) and/or StenB (30 μM) post-treatment for 0, 24, and 48 h. The proportions marked with (− ) were assigned to MMP (− ) cells. Data = mean ± SD (*n* = 3). Each treatment was assigned the connecting letters by software to judge significance. Connecting letters for different treatments without overlapping differed significantly (*p* < 0.05).

Following the StenB treatment, the MMP (−)(%) level in TNBC cells (HCC1937 and Hs578T) (Figure 7A) was raised, showing a higher MMP (−)(%) level in TNBC cells than in normal cells. Consequently, StenB effectively incurs mitochondrial oxidative stress in TNBC cells but not in normal cells.

Additionally, the potential role of oxidative stress in the StenB-induced MMP (−)(%) increase was evaluated by NAC. StenB demonstrated a higher MMP (−)(%) level in TNBC cells than NAC/StenB the treatment (Figure 7B). Accordingly, StenB incurs oxidative stress in TNBC cells by modulating oxidative stress.

### 2.8. DNA Damage Responses to StenB (TNBC vs. Normal Cells)

**Figure 8:**
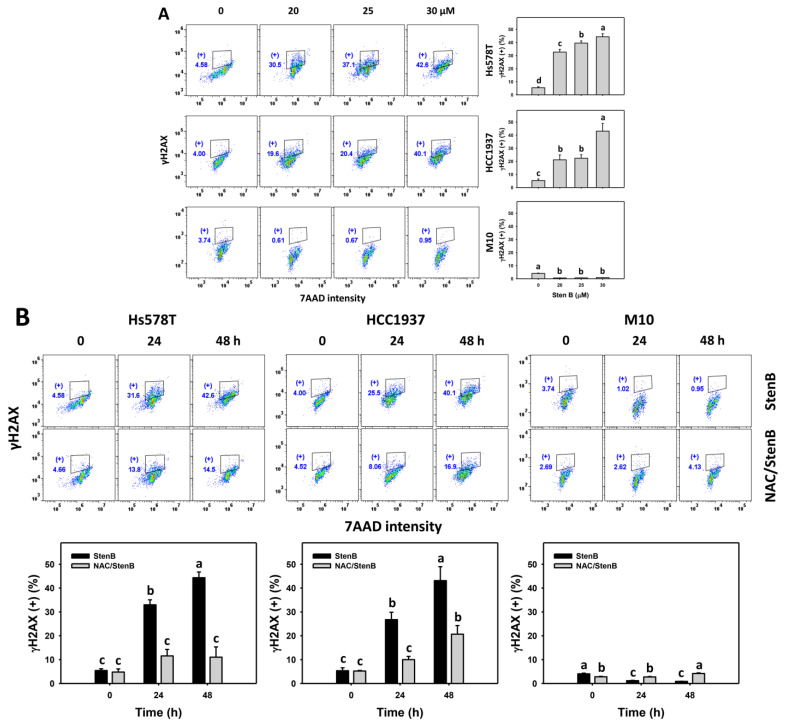
Effects of StenB on γH2AX intensity in TNBC and M10 cells. (**A**) γH2AX levels. TNBC and normal (M10) cells were treated with StenB for 48 h. (**B**) γH2AX levels of NAC and/or StenB. Cells received NAC pretreatment (10 mM, 1 h) and/or StenB (30 μM) post-treatment for 0, 24, and 48 h. The proportions marked with (+) were assigned to γH2AX (+) cells. Data = mean ± SD (*n* = 3). Each treatment was assigned the connecting letters by software to judge significance. Different letters above bars indicate significant differences (*p* < 0.05).

**Figure 9:**
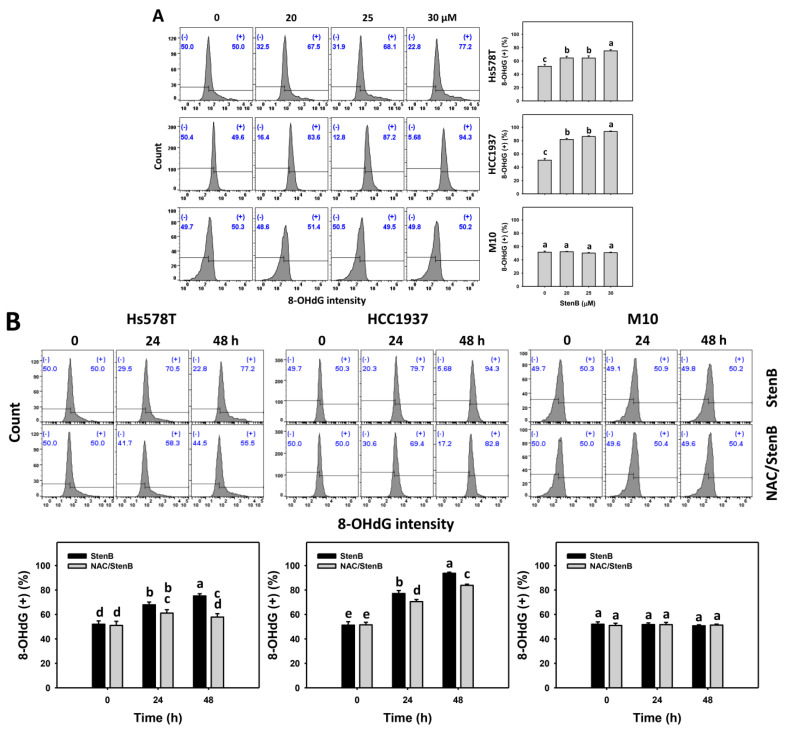
Effects of StenB on 8-OHdG intensity in TNBC and M10 cells. (**A**) 8-OHdG levels. TNBC and normal (M10) cells were treated with StenB for 48 h. (**B**) 8-OHdG levels of NAC and/or StenB. Cells received NAC pretreatment (10 mM, 1 h) and/or StenB (30 μM) post-treatment for 0, 24, and 48 h. The proportions marked with (+) were assigned to 8-OHdG (+) cells. Data = mean ± SD (*n* = 3). Each treatment was assigned the connecting letters by software to judge significance. Different letters above bars indicate significant differences (*p* < 0.05).

Following the StenB treatment, the γH2AX and 8-hydroxy-2-deoxyguanosine (8-OHdG) (+)(%) levels in TNBC cells (HCC1937 and Hs578T) (Figure 8A and Figure 9A) were raised, showing higher γH2AX and 8-OHdG (+)(%) levels in TNBC cells than in normal cells. Consequently, StenB effectively incurs DNA damage in TNBC cells but not in normal cells.

Additionally, the potential role of oxidative stress in the StenB-induced γH2AX and 8-OHdG (+)(%) increase was evaluated by NAC. StenB demonstrated higher γH2AX and 8-OHdG (+)(%) levels in TNBC cells than the NAC/StenB treatment (Figure 8B and Figure 9B). Accordingly, StenB incurs DNA damage in TNBC cells by modulating oxidative stress.

## 3. Discussion

*Boesenbergia*-plant-derived extracts and bioactive compounds show diverse biological effects. Recently, their antiproliferation effects have attracted much attention for use in cancer treatments, although there is a lack of knowledge regarding the detailed mechanism of their anticancer effects [17,19,20,21]. *B. stenophylla*, one of the Boesenbergia plants from Borneo, shows several well-known medicinal effects [7,17,25]. However, the anticancer effects of *B. stenophylla*-derived natural products remain unclear. The present study demonstrated that a new *B. stenophylla*-derived compound (StenB) [24] inhibited TNBC cells but not normal breast cells. To improve our understanding of the anticancer mechanism of StenB, several factors that exhibited differential responses between TNBC and normal cells were compared and are discussed here.

Although the antiproliferation of *B. stenophylla* was rarely reported, the anticancer effects of other Boesenbergia-plant-derived extracts and bioactive compounds have been mentioned, as follows: For comparison, rhizome extracts of *B. rotunda*, *B. pulchella* var. *attenuata*, and *B. armeniaca* show IC_50_ values of 51, 93, and 94.5 μg/mL, respectively, against breast cancer cells (MCF7) via a 72 h MTT assay [17]. Among these three species, only rhizome extracts of *B. rotunda* inhibit proliferation, showing an IC_50_ value of 66.5 μg/mL against TNBC cells (MDA-MB-231).

For comparison, the *Boesenbergia*-plant-derived bioactive compounds generally exhibit a higher drug sensitivity to anticancer effects than that of crude extracts. For example, *B. pandurate*-derived panduratin A showed an IC_50_ value of 3.75 μg/mL against non-TNBC cells (MCF-7) through a 72 h MTT assay [19]. *B. rotunda*-derived cardamonin showed IC_50_ values of 5.62 and 26.7 μg/mL against TNBC cells (MDA-MB-231) [26] and nasopharyngeal (HK1) [21] cancer cells, whereas the IC_50_ values of normal nasopharyngeal epithelial cells (NP69) were >200 μg/mL. This suggests cardamonin exhibits anticancer effects without severe side effects in normal cells.

Similarly, the IC_50_ values of StenB were 17.8 and 27.4 μM in TNBC cells (HCC1937 and Hs578T) through the 48 h ATP assay, while StenB had only a little effect on the viability of normal cells. Accordingly, the drug safety of StenB is validated in the cell model. The clinical drug cisplatin showed an IC_50_ value of 7.2 and 9.5 µM via the 48 h ATP assay for TNBC cells (HCC1937 and Hs578T), respectively. In this case, StenB is less sensitive compared to the TNBC treatment by cisplatin. However, cisplatin can potentially evoke side effects during clinical applications [27,28]. Additionally, the cell viability of the present study was conducted by mitochondrial-activity-dependent ATP assay. It warrants an alternative assay to evaluate cytotoxicity of StenB-treated TNBC cells using a protein-dependent assay, such as sulforhodamine B (SRB), which is not dependent on mitochondrial activity.

NAC reversely increases the cell viability of StenB-treated TNBC cells, indicating that oxidative stress is essential to trigger its antiproliferation function in TNBC cells (Figure 1C). Oxidative-stress-associated changes were further validated by several flow cytometry assays, such as ROS, MitoSOX, and MMP (Figure 6 and Figure 7). Notably, StenB-induced oxidative stress changes were higher in TNBC than in normal cells (Figure 6 and Figure 7), which might partly contribute to the preferential killing of TNBC cells but not of normal cells.

*Boesenbergia*-plant-derived extracts and bioactive compounds also exhibit a cell cycle disturbance and apoptosis-inducible function for the antiproliferation against cancer cells. However, the role of oxidative stress has rarely been investigated in previous studies [17,19,21]. For example, extracts of *B. rotunda* and *B. pulchella* var. attenuata caused moderate subGl accumulation and G2/M arrest in breast cancer MCF7 cells and induced dramatic sub G1 accumulation without G2/M arrest in MDA-MB-231 cells after 72 h treatments [17]. *B. pandurate*-derived panduratin-A-induced apoptosis of colon cancer cells occurred by increasing annexin V signals [19]. *B. rotunda*-derived cardamonin also exhibited antiproliferation, antimigration, G2/M arrest, and apoptosis-inducible effects in nasopharyngeal cancer cells [21]. Similarly, StenB promoted G2/M arrest and apoptosis-associated changes such as subG1 accumulation and annexin V intensity increases. StenB also activated caspase 8 and 9 signaling for triggering extrinsic and intrinsic apoptosis, and, finally, it converted this signaling to caspase 3 activation in TNBC cells (Figure 4). Notably, StenB-induced apoptosis and its signaling activations were higher in TNBC than in normal cells (Figure 3 and Figure 4), which might partly contribute to the preferential killing of TNBC cells but not of normal cells. Moreover, apoptosis does not completely cause the StenB-induced antiproliferation effects (Figure 1C), suggesting that other nonapoptotic cell deaths may participate in the StenB treatment of breast cancer cells.

The state of oxidative stress is a normal phenomenon in cellular functioning, especially during energy production. However, the overexpression of ROS, exceeding the tolerance of redox homeostasis, causes DNA damage [29], such as DNA double-strand breaks (γH2AX) [30,31] and oxidative DNA damage (8-OHdG) [32,33,34]. Consistently, StenB-promoted oxidative stress generates DNA damage (γH2AX and 8-OHdG) (Figure 8 and Figure 9). Notably, StenB-induced DNA damage was higher in TNBC than in normal cells, which contributes to the preferential killing of TNBC cells compared to normal cells.

Moreover, all oxidative-stress-associated changes were upregulated by StenB. NAC experiments proved that the downregulation of oxidative-stress-alleviated StenB-induced cell cycle redistribution, oxidative stress, and DNA damage in TNBC cells. Consequently, StenB drives the antiproliferation mechanism of anticancer effects through oxidative stress modulation.

*B. stenophylla* exhibits medicinal properties [7,23] and may have drug safety for human treatment. The drug safety of the bioactive compound of *B. stenophylla* in the example of StenB has been validated by the cell model that StenB shows preferential antiproliferation to breast cancer but not for normal cells. However, the present study mainly provided 2D cell experiments without 3D analysis, such as mammosphere formation and its associated changes [35,36]. Consequently, the detailed functions of StenB need further investigation. Moreover, in vivo biodistribution, anticancer function, and biosafety of StenB remains unclear. Recently, molecular docking analyses of major bioactive compounds of *B. rotunda* have been applied to identify its potential target [37]. Understanding drug targeting can specifically improve cancer therapy. Notably, the limitation of the present study is the absence of in vivo antibreast cancer function, biosafety, and target identification for StenB. It warrants an advanced assessment of the in vivo antibreast cancer effects of StenB in the future.

## 4. Materials and Methods

### 4.1. Preparation of StenB and Reagents

*B. stenophylla* was identified and collected for StenB extraction as described in our previous work [24]. The purity of StenB was >95% as determined by NMR spectrum examination [24] and provided in the Supplementary Figures S1 and S2, respectively. StenB was dissolved in DMSO for cell experiments at 0.1%. *N*-acetylcysteine [38,39,40] (Sigma-Aldrich, St. Louis, MO, USA) was dissolved in 1X PBS for pretreatment (10 mM, 1 h) to evaluate the involvement of oxidative stress in StenB-treated cells. Z-VAD-FMK (ZVAD) was dissolved in DMSO for pretreatment (100 μM, 2 h) to assess the participation of apoptosis in StenB-treated cells. Cisplatin (Selleckchem.com; Houston, TX, USA) was freshly dissolved in 1X PBS before experiments.

### 4.2. Cell Cultures and Viability

Cell lines for TNBC (HCC1937 and Hs578T from ATCC (Manassas, VA, USA)) were used to test the antiproliferation effects of StenB. The drug safety of StenB was inspected by testing a nonmalignant normal breast epithelial cell line (H184B5F5/M10 (M10)) (Bioresource Collection and Research Center, Hsinchu, Taiwan). Except for M10 cells (alpha medium), the medium for breast cancer cells was DMEM/F12 (3:2) (Gibco, Grand Island, NY, USA) enriched with 10% fetal bovine serum and P/S antibiotics.

An ATP detection kit (PerkinElmer Life Sciences, Boston, MA, USA) was applied to detect cellular ATP content, indicating cell viability [41,42]. In brief, 12.5 μL of the substrate (D-luciferin and luciferase) was added to the cell lysate (50 μL) and reacted for 5 min in the dark. Finally, the signal was monitored by a microplate luminometer (Berthold Technologies GmbH and Co., Bad Wildbad, Germany).

### 4.3. Cell Cycle

Next, 75% ethanol-fixed cells were suspended in 7AAD (1 μg/mL) reagent (Biotium, Hayward, CA, USA) for a 20 min incubation [41]. The DNA intensity of fixed cells was assessed with the flow cytometer (Guava easyCyte, Luminex, Austin, TX, USA), and the cell phases were analyzed via its program.

### 4.4. Apoptosis and Caspases 3, 8, and 9

Annexin V/7AAD double-staining [43,44] was carried out with the routine apoptosis assay. With a minor modification, cells were stained with annexin V-FITC/7AAD (1:1000/1 μg/mL) [43] (Strong Biotech, Taipei, Taiwan) for 1 h. Their signals were inspected by a flow cytometer. The apoptosis proportions within the regions of annexin V (+)/7AAD (+ or −) intensity were assigned to apoptotic (+) cells.

In addition to annexin V/7AAD, apoptosis was also detected regarding caspase (Cas) activity, such as caspases 3, 8, and 9. According to the user’s manual, the activities of these caspases were inspected via flow-cytometry-based OncoImmunin’s-specific peptides for caspases 3, 8, and 9 (Gaithersburg, MD, USA) [45]. Only the activated caspases 3, 8, and 9 can cut the peptide substrates and become fluorescent molecules. These activated signals were assessed by a flow cytometer.

### 4.5. Oxidative Stress

Three types of oxidative stress biomarkers were assessed, including ROS, MitoSOX, and MMP. According to the user’s manual, their matched probes, such as 2′,7′-dichlorodihydrofluorescein diacetate (H_2_DCFDA) [46] (Sigma-Aldrich), MitoSOX™ Red [47] (Thermo Fisher Scientific, Carlsbad, CA, USA), and DiOC_2_(3) (Invitrogen, San Diego, CA, USA) [48], were chosen to detect the ROS, MitoSOX, and MMP, respectively. After the oxidative stress reaction, these probes become fluorescent molecules. These activated signals were assessed by a flow cytometer.

### 4.6. DNA Damages

Two types of DNA damage were detected and assessed through antibody-based analyses [47], including markers for DNA double-strand breaks (γH2AX) [30,31] and oxidative DNA damage (8-OHdG) [32,33]. Briefly, 75% ethanol-fixed cells were processed with γH2AX primary antibody/secondary antibody/7AAD (5 μg/mL) treatments [40] or the 8-OHdG primary antibody [47] (Santa Cruz Biotechnology, Santa Cruz, CA, USA) to detect the γH2AX and 8-OHdG levels, respectively. These activated signals were assessed by a flow cytometer.

### 4.7. Statistical Analysis

Significance for multicomparison was assessed via the post hoc test with ANOVA analysis (JMP software 12 (SAS Institute Inc., Cary, NC, USA)). Each treatment was assigned the connecting letters by software to judge significance. Treatments with different letters above the bars differed significantly.

## 5. Conclusions

The present study firstly explored the antiproliferative functions of StenB with low cytotoxicity effects on normal cells. This characteristic of preferential antiproliferation in StenB was associated with the upregulation of more cell cycle disturbances, apoptosis, oxidative stress, and DNA damage in TNBC than in normal cells. Both extrinsic and intrinsic apoptotic signaling was triggered by TNBC cells. Through the NAC pretreatment, all the characteristics of preferential antiproliferation of StenB in TNBC cells were validated to depend on oxidative stress. In conclusion, StenB is a potential *B. stenophylla*-derived natural product for treating TNBC that works by modulating oxidative-stress-associated responses.

## Data Availability

All data are contained within the article.

## Supplementary Materials

The supporting information can be downloaded at: https://www.mdpi.com/article/10.3390/ijms24097751/s1.
