# Supplementary material for: Boesenbergia stenophylla-Derived Stenophyllol B Exerts Antiproliferative and Oxidative Stress Responses in Triple-Negative Breast Cancer Cells with Few Side Effects in Normal Cells
Source: Int J Mol Sci. 2023 Apr 24;24(9):7751. doi: 10.3390/ijms24097751 (PMC10178828; doi:10.3390/ijms24097751)
Supplement: Supplementary file 1 [file ijms-24-07751-s001.zip › ijms-2342220-supplementary.pdf]

**SUPPLEMENTARY MATERIAL**

## Stenophyllol B Spectral

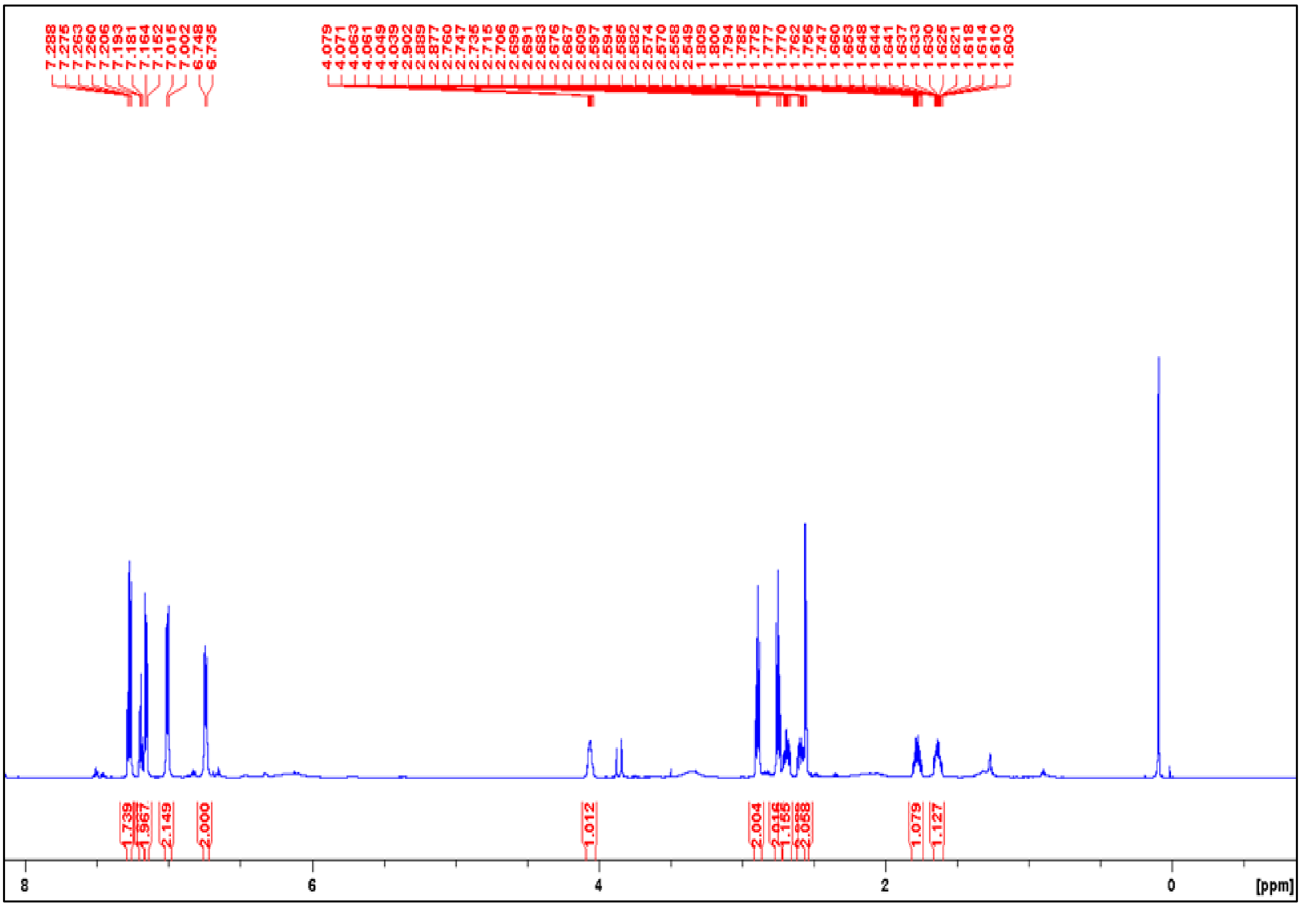


Figure **S1**. $^{1}$H NMR spectrum ($CDCl_3$, 600 MHz) of Stenophyllol B.

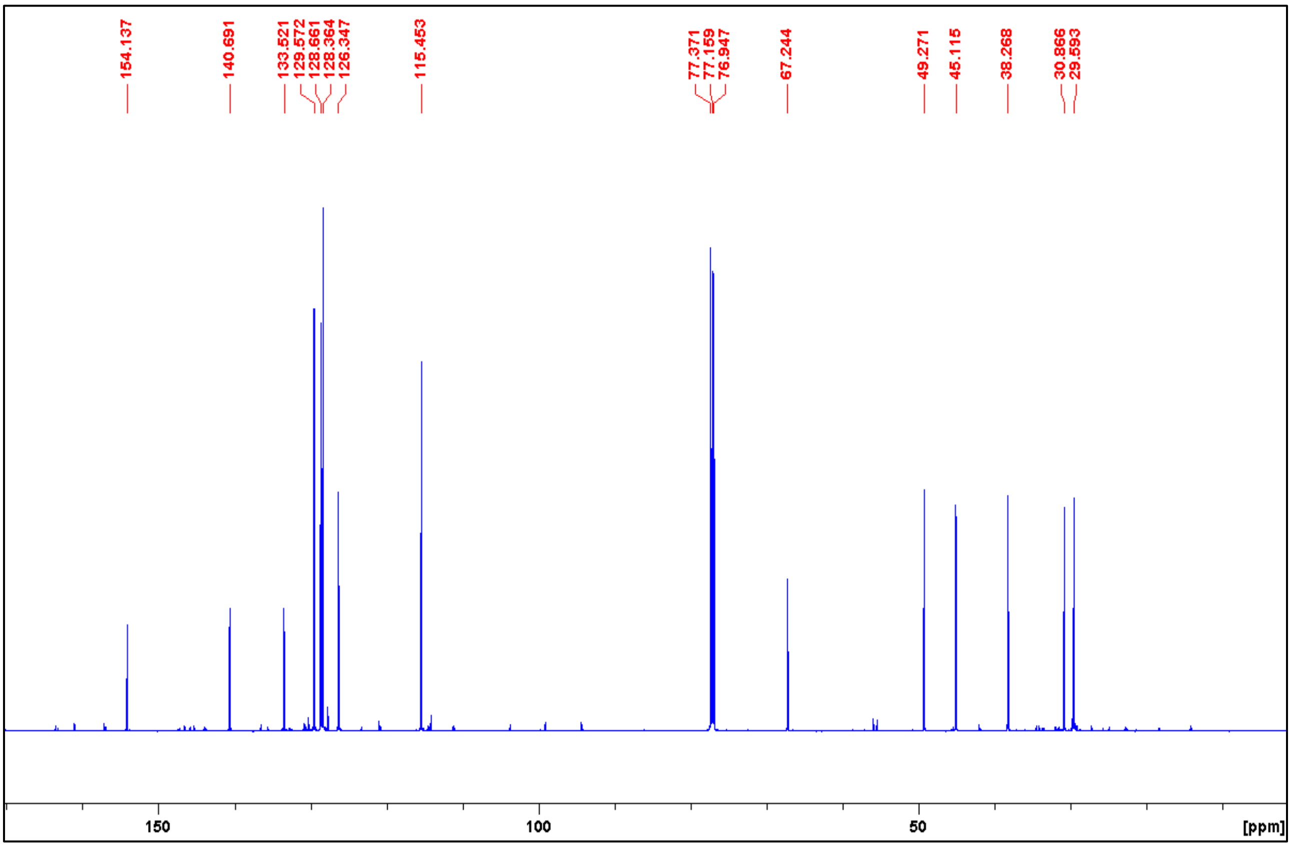


Figure **S2**. $^{13}C$ NMR spectrum ($CDCl_3$, 150 MHz) of Stenophyllol B.
